# Protective Mechanism of Hydrogen Sulfide against Chemotherapy-Induced Cardiotoxicity

**DOI:** 10.3389/fphar.2018.00032

**Published:** 2018-01-26

**Authors:** Shuxu Du, Yaqian Huang, Hongfang Jin, Tianyou Wang

**Affiliations:** ^1^Department of Pediatrics, Beijing Shijitan Hospital, Capital Medical University, Beijing, China; ^2^Department of Pediatrics, Peking University First Hospital, Beijing, China; ^3^Hematology/Oncology Center, National Center for Children's Health, Beijing Children's Hospital, Capital Medical University, Beijing, China

**Keywords:** apoptosis, cardioprotection, cardiotoxicity, chemotheray, hydrogen sulfide, inflammation, oxidative stress

## Abstract

Over the past few decades, the number of long term survivors of childhood cancers has been increased exponentially. However, among these survivors, treatment-related toxicity, especially cardiotoxicity, is becoming the essential cause of morbidity and mortality. Thus, preventing the treatment-related adverse effects is important to increase the event free survival during the treatment of cancer in children and adolescents. Accumulating evidence has demonstrated that hydrogen sulfide (H_2_S) exerts a protective role on cardiomyocytes through a variety of mechanisms. Here, we mainly reviewed the cardioprotective role of H_2_S in the chemotherapy, and emphatically discussed the possible mechanisms.

## Introduction

Over the past several decades, survival rates of children and adolescents with cancer have been significantly improved (Madhusoodhan et al., 2016). However, chemotherapy-induced cardiovascular complications are becoming main causes of morbidity and mortality among these long-term survivors (Zamorano et al., 2016; Hutchins et al., 2017; Lopez-Fernandez et al., 2017). Many anticancer drugs, such as doxorubicin (or adriamycin), mitoxantrone, cyclophosphamide, cisplatin and 5-fluorouracil, are well-known as chemotherapeutic agents for treating leukemia, lymphomas, neuroblastoma, and sarcoma (Govender et al., 2014; Meserve et al., 2014; Polk et al., 2014; Damiani et al., 2016; Dugbartey et al., 2016; Giza et al., 2017). However, the dose-dependent and independent cardiotoxic effects would hinder their clinical usage and affect the long-term life quality of all survivors (Meserve et al., 2014; Polk et al., 2014; Damiani et al., 2016; Dugbartey et al., 2016; Giza et al., 2017). Thus, the cardio-oncologists had to explore new preventive agents and strengthen the heart protection during or after treatment of patients with cancer (Albini et al., 2010; Lenneman and Sawyer, 2016).

**Figure 1:**
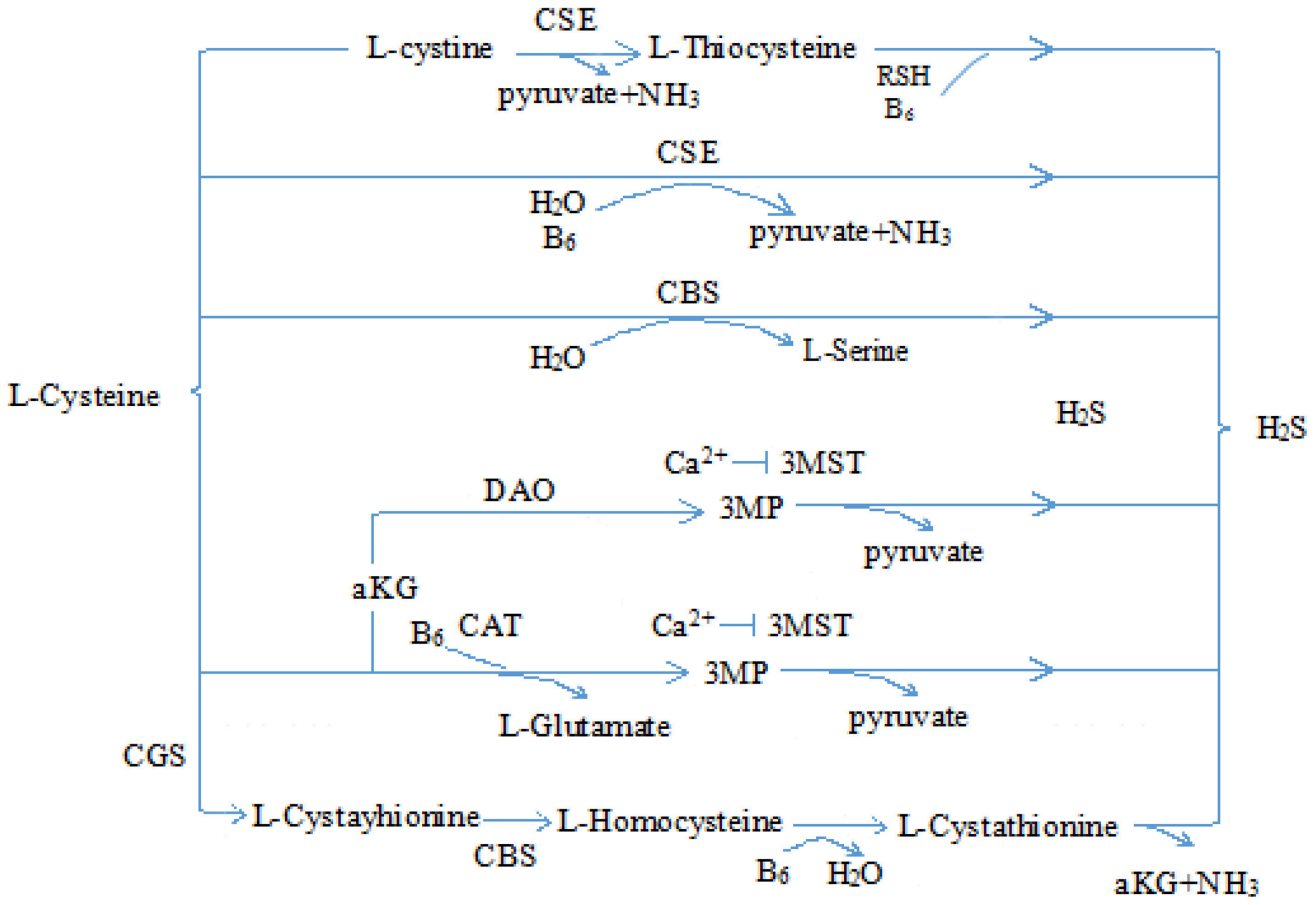
Physiological production of hydrogen sulfide. CSE, cystathionine-γ lyase; CBS, cystathionine-β synthase; 3-MST, 3-mercaptopyruvate sulfurtransferase; CAT, cysteine aminotransferase; 3MP, 3-mercaptopyruvate; αKG, α-ketobutyrate glutamate; H_2_S, hydrogen sulfide.

Hydrogen sulfide (H_2_S) is a pollutant gas with strong rotten-egg odor. Accumulating research has shown that H_2_S is endogenously generated from *L*-homocysteine and *L*-cysteine in human and animal organisms, and cystathionine-γ lyase (CSE) is the most important enzyme in cardiovascular tissues (Kimura, 2011; Olson and Straub, 2016; Tabibzadeh, 2016) (see Figure 1). Once produced, H_2_S is broken down rapidly and participates in many pathophysiological processes (Tang et al., 2006; Du et al., 2011, 2014; Sun et al., 2011; Altaany et al., 2014; Yang and Wang, 2015; Bian et al., 2016; Cao and Bian, 2016; Panthi et al., 2016). More importantly, recent evidence shows that H_2_S may play cytoprotective role in heart diseases (Shen et al., 2015; Liu et al., 2016a; Szabo, 2016; Huang et al., 2017). Here, we mainly reviewed the cardioprotective role of H_2_S in chemotherapy and its possible mechanisms.

## Chemotherapy induced cardiotoxicity

With the longer survival of cancer patients, larger number of cancer patients will be potentially at the risk of early cardiovascular morbidity and mortality resulting from chemotherapy (Curigliano et al., 2016). Cardiotoxicity may occur during or after cancer therapy, exhibiting various symptoms including asymptomatic decreases in left ventricular ejection fraction and life-threatening congestive heart failure (CHF) (Lipshultz et al., 2015). Generally, chemotherapy-induced cardiotoxicity is broadly divided into two types, type I and type II. Type I is more severe and thought to be irreversible, which is classically associated with anthracycline drugs such as doxorubicin, daunorubicin, epirubicin, and idarubicin (Lipshultz et al., 2013; Ai et al., 2014; Zamorano et al., 2016). While, type II toxicity is thought to be less severe and potentially reversible, and associated with non-anthracycline drugs (O'Hare et al., 2015; Zamorano et al., 2016).

The most widely accepted mechanisms for anthracycline actions are related to the fact that anthracyclines interfere redox cycling, which results in DNA damage due to the reactive oxygen species (ROS) production (O'Hare et al., 2015; McGowan et al., 2017), then triggers a series of oxidative stress, lipid peroxidation, mitochondrial dysfunction, apoptosis and dysregulation of autophagy successively and/or; simultaneously (Geisberg and Sawyer, 2010; Menna et al., 2012; Govender et al., 2014; Mitry and Edwards, 2016; McGowan et al., 2017). Doxorubicin, in particular, directly damages mitochondria of cardiomyocytes, produces ROS, breaks the delicate balance of the ROS-generating system and antioxidant defense system, and leads to the oxidative stress (Geisberg and Sawyer, 2010; Varga et al., 2015). In general, oxidative stress is demonstrated to be one of the major potential causes and ultimately leads to cardiomyopathy and heart failure (Menna et al., 2012; Varga et al., 2015; Tocchetti et al., 2017). Furthermore, oxidative stress can cause cellular hypertrophy, extracellular matrix (ECM) remodeling, cardiac contraction impairment, and cardiomyocyte death and autophagy (Lipshultz et al., 2013; Angsutararux et al., 2015; Zamorano et al., 2016).

On the other hand, many non-anthracycline drugs, such as alkylating agents (cyclophosphamide), platinum agents (cisplatin), antimetabolites (5-fluorouracil), and monoclonal antibodies (imatinib and bevacizumab) (Dirks-Naylor, 2013; Madeddu et al., 2016; Hrynchak et al., 2017), can cause type II cardiotoxicity, which is not dose-dependent and always reversible when the drug is stopped (Higgins et al., 2015; Madeddu et al., 2016), and the prognosis is more favorable than that induced by anthracyclines (Hrynchak et al., 2017). The mechanisms for non-anthracycline-induced cardiotoxicity vary broadly among several chemotherapeutic agents, and the most common mechanism is directly damaging myocardial cells or causing pericardium inflammation (Albini et al., 2010). For example, paclitaxel directly damaged cardiomyocyte targeting on subcellular organelles, and resulted in bradycardia, intracardiac block, or arrhythmia in cancer patients (Guo and Wong, 2014). 5-fluorouracil has direct impact on the vascular endothelium and protein kinase C, and finally leads to the endothelium-independent vasoconstriction and coronary constriction (Panis et al., 2012). Similar to doxorubicin, cisplatin can impair the antioxidant defense system of the cells, causing oxidative stress and injuring renal and cardiac tissues (Schlitt et al., 2014; Layoun et al., 2016). Moreover, cisplatin also enhanced the platelet aggregation and thromboxane formation, activated arachidonic acid pathways (Dugbartey et al., 2016), led to vascular toxicity, such as acute coronary artery thrombosis, and increased the long-term cardiovascular risk (Herrmann et al., 2016).

Since cardiovascular injury is the fatal adverse outcome of anticancer treatment, safer anticancer drugs should be developed, and newer chemotherapeutic regimens should be considered in the management of cancer survivors.

## Role of H_2_S in chemotherapy-induced cardiotoxicity

It has been proved that H_2_S can protect heart from myocardial infarction, ischemia-reperfusion injury and heart failure (Khatua et al., 2013; Xie et al., 2015), regulate cardiac function and structure, and attenuate the damage of myocardial cells (Shen et al., 2015). Recent studies have revealed that H_2_S can protect cardiomyocyte toxicity induced by doxorubicin (see Table 1). Su et al. (2009) first reported that after treatment rats with adriamycin (ADR), greater size and smaller number of cardiomyocytic mitochondria were found, and H_2_S levels in plasma and myocardium were decreased simultaneously. However, after administration of H_2_S donor, sodium hydrosulfide (NaHS), the left ventricular developed pressures were elevated and the morphological alterations of myocardium were ameliorated, and then the cardiac function was markedly improved. The protective effect of H_2_S on the doxorubicin-induced cardiac injury was verified in another rat model with doxorubicin-induced cardiomyopathy (Yu et al., 2017). In *in vitro* experiments, Wang et al. demonstrated that exogenous H_2_S could protect against DOX-induced cardiotoxicity partly through the inhibition of endoplasmic reticulum (ER) stress (Wang et al., 2012). Another research group further observed that exogenous H_2_S played protective roles in H9c2 cells through inhibiting p38 MAPK pathway (Guo et al., 2013a). They pretreated H9c2 cells with either NaHS or p38 MAPK inhibitor, and noticed that the viability of cells was increased, apoptotic cells counting and ROS generation were decreased significantly, and the doxorubicin-induced toxicities were significantly ameliorated. Moreover, Liu et al. found that exogenous H_2_S attenuated DOX-induced cardiotoxicity by inhibiting calreticulin expression and activated extracellular signal-regulated kinase (ERK) 1/2 in H9c2 cardiomyocytes (Liu et al., 2015a, 2016b). Overall, numerous researches demonstrated that H_2_S could protect against DOX-induced toxicity in cardiomyocytes.

**Table 1:** The anticancer drugs-caused cardiac injury was antagonized by H_2_S.

**Drug/class of drugs**	**Cardiac toxic effect limited by H_2_S**	**Mechanism of action**
Adriamycin (Su et al., 2009)	H_2_S improves the impairment of cardiac function and alleviates the cardiac pathological change in adriamycin-treated rats.	Reduces lipid peroxidation, increases the activities of antioxidant enzyme, and therefore inhibits oxidative stress injury.
Doxorubicin (Wang et al., 2012)	H_2_S improves cellular survival in the doxorubicin-treated H9c2 cardiac cells.	Inhibits the endoplasmic reticulum stress.
Doxorubicin (Guo et al., 2013a)	H_2_S attenuates the doxorubicin-induced inflammation and cytotoxicity in H9c2 cardiac cells.	Depresses the p38 MAPK/NF-κB pathway.
Doxorubicin (Guo et al., 2013b)	H_2_S protects against doxorubicin-induced cytotoxicity, apoptosis, mitochondrial damage and oxidative stress in H9c2 cardiac cells.	Inhibits the p38 MAPK pathway.
Doxorubicin (Liu et al., 2015a,b, 2016a,b)	H_2_S protects against doxorubicin-induced cytotoxicity and apoptosis in H9c2 cardiac cells.	1. Inhibits calreticulin expression. 2. Suppresses reactive oxygen species-activated extracellular signal-regulated kinase 1/2 pathway. 3. Restores the imbalance between anti-apoptotic protein bcl-2 and pro-apoptotic protein bax. 4. Inhibits peroxiredoxin III expression. 5. Activates PI3K/Akt pathway, enhances the phosphorylation of FoxO3a and then reduces the nuclear translocation of FoxO3a.
Doxorubicin (Yu et al., 2017)	H_2_S protects against doxorubicin-induced dilated cardiomyopathy in rats.	1. Activates Nrf2 signaling to reduce doxorubicin-induced oxidative stress. 2. Activates PI3K/Akt pathway to exert antiapoptotic effects.
Doxorubicin (Zhang et al., 2011)	S-diclofenac, a novel H_2_S-releasing derivative of diclofenac, significantly ameliorates doxorubicin-related cardiac injury and cardiac dysfunction, and improves the survival rate of mice with doxorubicin-induced cardiomyopathy.	1. Reverses the cardiac gap junction remodeling via suppressing the activation of JNK pathway. 2. Inhibits the oxidative stress and inflammation in the mouse heart.
Doxorubicin (Chegaev et al., 2016)	H_2_S-DOXO, an H_2_S releasing DOXO derivative, protects against DOXO-induced cytotoxicity in H9c2 cardiac cells.	Reduces the amount of ROS produced by DOXO.
Doxorubicin (Wu et al., 2016)	SPRC, a producing agent of endogenous H_2_S, prevents doxorubicin-induced cardiac cytotoxicity *in vitro* and *in vivo*.	Activates the gp130/STAT3 pathway, and then inhibits apoptosis and oxidative stress, finally leads to antagonizing mitochondrial dysfunction and intracellular Ca^2+^ overload in the doxorubicin-treated mice and H9c2 cells.

## Protective mechanisms of H_2_S in chemotherapy-induced cardiotoxicity

**Figure 2:**
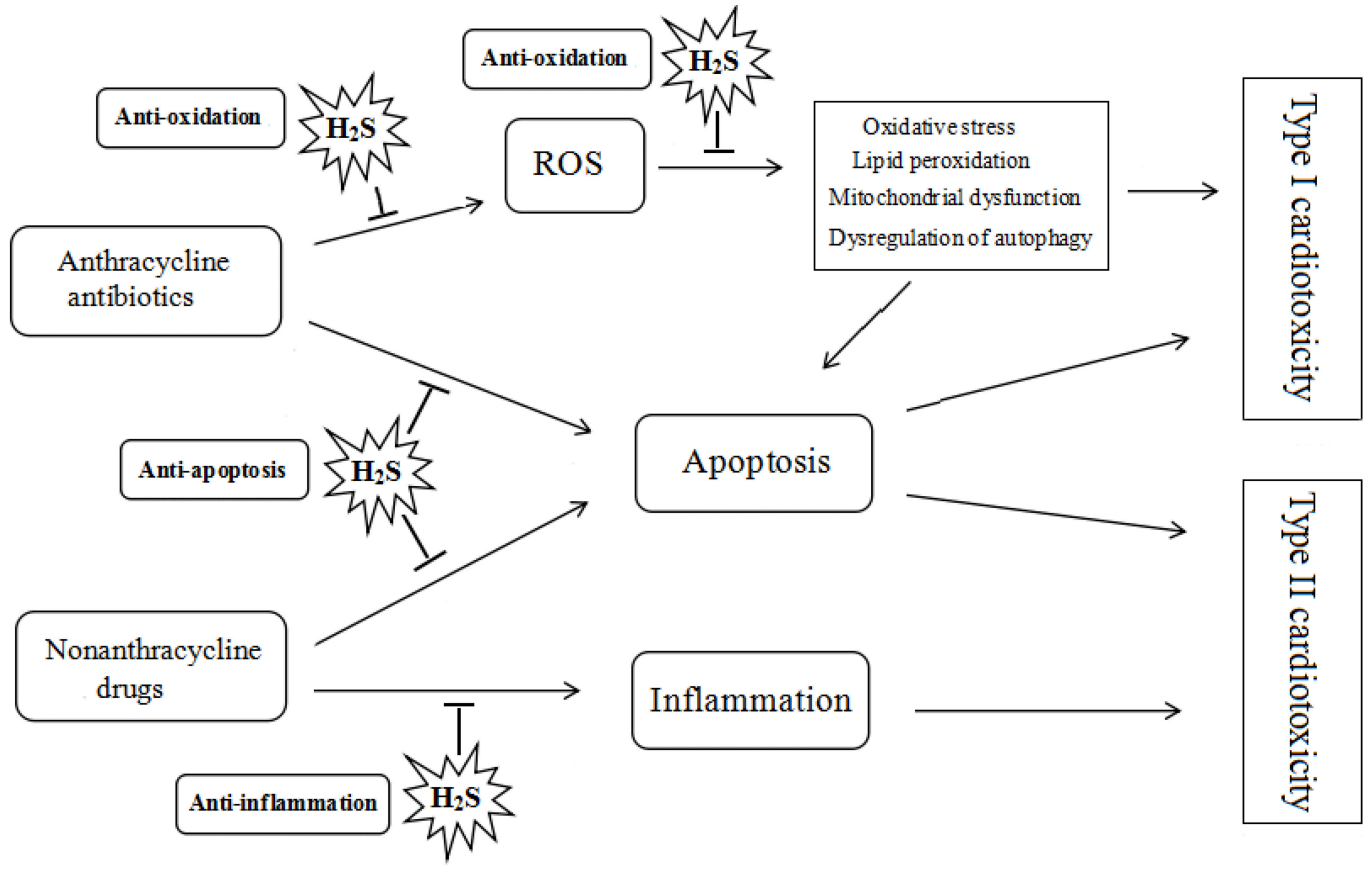
Protective role of hydrogen sulfide on chemotherapy-induced cardiotoxicity. ROS, reactive oxygen species; H_2_S, hydrogen sulfide.

It has been demonstrated that H_2_S plays a protective role in the pathogenesis and development of heart diseases. The effect of H_2_S on the chemotherapy-induced cardiotoxicity may be mediated by a diverse array of cellular and molecular signals. Many researches have identified that H_2_S can attenuate doxorubicin-induced cardiotoxicity through inhibiting oxidative stress, reducing apoptosis and attenuating inflammatory responses (see Figure 2).

### Anti-oxidation

As mentioned before, doxorubicin can injure myocardial cells by elevating the generation of ROS, destroying the balance of the ROS-generating system and antioxidant defense system, and causing oxidative stress (Angsutararux et al., 2015). However, it has been proved that H_2_S is a strong antioxidant and plays a protective role in the cardiac system (Salloum, 2015; Shen et al., 2015).

As demonstrated previously by Su et al., H_2_S could reduce lipid peroxidation, increase the activity of antioxidant enzyme, and inhibit oxidative stress injury in the pathogenesis of adriamycin-induced cardiomyopathy (Su et al., 2009). They observed that after treating rats with adriamycin, the level of thiobarbituric acid reactive substance (TBARs) was increased, while the activities of superoxide dismutase (SOD) and glutathione peroxidase (GSH-Px) in plasma and myocardium were decreased. However, after the administration of NaHS, the myocardial TBARs content was decreased, and the activities of SOD and GSH-Px were increased. Furthermore, Yu et al. discovered that H_2_S suppressed the doxorubicin-induced oxidative stress through activating the Nrf2 signaling pathway and then upregulating the expressions of its downstream genes antioxidant proteins NQO1 and GCLM (Yu et al., 2017). In addition, Wang et al. reported that H_2_S could protect H9c2 cells through inhibiting ER stress (Wang et al., 2012). After exposure to doxorubicin, they found that the CSE expression and activity in H9c2 cells were inhibited obviously, however, the ER stress-related proteins, glucose-regulated protein 78 (GRP78) and C/EBP homologous protein (CHOP), were upregulated obviously. Furthermore, after pretreatment with NaHS before doxorubicin exposure, the GRP78 and CHOP overexpression by H9c2 cells, oxidative stress and cytotoxicity were suppressed markedly. Moreover, Liu et al. observed that pretreatment with NaHS prior to doxorubicin exposure could increase the cell viability and decrease the intracellular accumulation of ROS in H9c2 cells (Liu et al., 2015a). They found that after the exposure of H9c2 cells to doxorubicin, the phosphorylated ERK1/2 levels were upregulated, however, after pretreatment with NaHS, phosphorylated ERK1/2 levels were reduced. Moreover, they observed that selectively inhibiting ERK1/2 could further obtain the above-mentioned effects of H_2_S. Liu and his colleagues also found that doxorubicin could induce cytotoxicity through increasing the peroxiredoxin III expression. They pretreated H9c2 cells with NaHS and found that the cell viability was increased, cell apoptosis attenuated, and the peroxiredoxin III overexpression reversed (Liu et al., 2016a). Therefore, H_2_S might play an antioxidant role in doxorubicin-induced cardiotoxicity via inhibiting ROS generation, ER stress-related proteins levels, peroxiredoxin III expression, and ERK1/2 phosphorylation and activating Nrf2 and its downstream pathway.

### Anti-apoptosis

It has been proved that doxorubicin-induced cardiomyocyte apoptosis is stimulated by mitochondrial pathway (Sun et al., 2016), rather than the death receptor pathway (Zhao and Zhang, 2017). Accumulating evidence has shown that the increased oxidative stress could directly or indirectly activate several signaling pathways, which further leads to the cardiomyocyte apoptosis (Sun et al., 2016). Sun and his colleagues summarized that ERK was initially phosphorylated in the H9c2 cells after the exposure to doxorubicin, and then the tumor suppressor gene p53 was activated. After the gene p53 was translocated to the nucleus, expression of p53 was increased, and the Bcl-2 family genes were activated, triggering the collapse of the mitochondrial membrane potential, cytochrome c release, activation of caspase-9 and caspase-3, and, ultimately, cell death via apoptosis (Sun et al., 2016).

Based on previous studies, H_2_S might play anti-apoptotic role in doxorubicin-induced cardiotoxicity. As mentioned before, Liu et al. demonstrated that H_2_S could downregulated phosphorylated ERK1/2 expression and decreased H9c2 cells apoptosis (Liu et al., 2015b). They also observed that pretreating H9c2 cells with NaHS prior to doxorubicin exposure, the anti-apoptotic protein Bcl-2 was upregulated, however, the pro-apoptotic protein Bax was downregulated (Liu et al., 2015b). Furthermore, Liu and his colleagues also observed that H_2_S could protect H9c2 cardiac cells through the PI3K/Akt/FoxO3a pathway. They pretreated H9c2 cardiomyocyte with NaHS for 30 min prior to doxorubicin exposure, and observed that the phosphorylation of Akt and FoxO3a, the FoxO3a nuclear localization, and the apoptosis of H9c2 cells were markedly attenuated. Moreover, they pretreated H9c2 cells with LY294002, a selective inhibitor of PI3K/Akt, and found the protective effect of H_2_S was reversed (Liu et al., 2016b). Additionally, Guo et al. also observed that the level of phosphorylated (p)-p38 MAPK was increased after exposure to doxorubicin, however, if pretreating H9c2 cells with either NaHS or an inhibitor of p38 MAPK, the H9c2 cardiomyocyte injuries could be ameliorated (Guo et al., 2013a). In *in vivo* experiment, restoration of the decreased PI3K/Akt pathway was observed in the myocardial tissues of rat administrated with doxorubicin + H_2_S, which was concomitant with the decrease in the cell apoptosis in the myocardial tissues. Furthermore, PI3K knockdown blocked the anti-apoptotic effect of H_2_S on the doxorubicin-treated primary rat cardiomyocyte in *in vitro* experiment (Yu et al., 2017).

These findings presented the evidence that H_2_S could play anti-apoptotic role in doxorubicin-induced cardiomyocyte through many signaling pathways.

### Anti-inflammation

Cardiac inflammation is well-known to participate in the pathogenesis of doxorubicin-induced cardiotoxicity. Guo and his colleagues have proved that the p38 MAPK/NF-κB pathway is an important signaling mechanism in the induction of doxorubicin-induced inflammation in H9c2 cardiomyocytes (Guo et al., 2013b). They observed that after the treatment of H9c2 cells with doxorubicin, cell viability was reduced and an inflammatory response was stimulated, demonstrated by an increasing production of interleukin-1β (IL-1β), IL-6, and tumor necrosis factor-α (TNF-α). They also found that the phosphorylated p38 MAPK and NF-κB p65 subunit were overexpressed. Toldo and his collegues demonstrated that Na_2_S played cardioprotective effects through miR-21-dependent attenuation of ischemic and inflammatory injury in cardiomyocytes (Toldo et al., 2014), which supported the anti-inflammatory effect of H_2_S during the development of cardiac injury. Therefore, Guo and his colleges verified the hypothesis that H_2_S played an anti-inflammatory effect on the doxorubicin-induced cardiotoxicity (Guo et al., 2013b,c). They pretreated H9c2 cells with NaHS for 30 min before its exposure to doxorubicin, and observed that doxorubicin-induced phosphorylation and nuclear translocation of NF-κB p65 subunit were markedly ameliorated, and inflammatory responses induced by doxorubicin were also significantly attenuated. Moreover, Zhang and colleagues observed the cardioprotective effect of S-diclofenac, a H_2_S-releasing derivative. They injected a single dose of doxorubicin (15 mg/kg, i.p.) to male C57BL/6J mice, and then S-diclofenac (25 and 50 μmol/kg, i.p.) was given for 2 weeks, and observed that S-diclofenac could play dose-dependent anti-inflammatory roles in this mice model (Zhang et al., 2011).

All the above mentioned researches demonstrated that H_2_S could play anti-inflammatory role in doxorubicin-induced cardiomyocyte injury.

## Clinical prospect of H_2_S as cardiac protective agents

As a signaling molecule, the fields of H_2_S physiology and pharmacology have been rapidly growing in recent years (Kimura, 2015; Wallace and Wang, 2015; Xie et al., 2015). H_2_S releasing agents (also known as H_2_S donors), such as H_2_S gas, sulfide salts, and garlic-derived sulfur compounds, have been widely used not only as research tools, but also as therapeutic agents (Zhao et al., 2014, 2015; Bełtowski, 2015; Wallace et al., 2017). For example, H_2_S-releasing drugs, such as SG1002 and ATB-346, have shown considerable promise for clinical trials (Wallace et al., 2017). Another H_2_S slow-release donor, protein-nanoemulsions (BAD-NEs), a novel formulation of diallyl disulfide (DADS) and α-linolenic acid (ALA), was studied by Ciocci et al. (2016). They found that BAD-NEs were able to regulate the ERK1/2 pathway, induce apoptosis, restrain cell cycle at G0/G1 phase, and inhibit the proliferation of different human cancer cell lines including MCF-7 breast cancer and HuT 78 T-cell lymphoma cells.

Khatua and his colleagues reviewed that dietary garlic could play cardioprotective effects by the generation of H_2_S and NO in cardiomyocytes and endothelial cells. Garlic could extenuate doxorubicin-induced cardiotoxicity by reducing lipid peroxidation, inducing cardiac endogenous antioxidants, inhibiting histone deacetylase and cytochrome P450, modulating Akt signaling pathways and regulating ion channels (Khatua et al., 2013). S-propargyl-cysteine (SPRC) is a producing agent of endogenous H_2_S, and possesses cardioprotective efficacy. Wu and his colleagues have demonstrated that SPRC can stimulate the activation of STAT3 via gp130-mediated transduction tunnel (Wu et al., 2016). In doxorubicin-induced cardiotoxicity, SPRC could enhance cell viability, restore expression of gp130/STAT3-regulated downstream genes, inhibit apoptosis and oxidative stress, and antagonize mitochondrial dysfunction and intracellular Ca(2+) overload. This will offer an innovative molecular basis and therapeutic strategy of H_2_S donor for the treatment of heart failure. Moreover, Chegaev et al. reported a series of new doxorubicin derivatives (H_2_S-DOXOs), which combined doxorubicin with appropriate H_2_S donor substructures. These H_2_S-releasing doxorubicins were safe and effective at 5 μM concentration on H9c2 cells, without any toxicity (Chegaev et al., 2016). They observed that a few compounds even could trigger high activity on the cancer cells and might be used to take the place of naked doxorubicin in the future. Once applied to clinical practice, these H_2_S-DOXO compounds will bring great benefits to solid cancer and leukemia patients.

In addition to the cardioprotective effect of H_2_S on the antineoplastic drug-induced cytotoxicity, its impact on the effectiveness of antineoplastic drug should be considered when the clinical prospect of H_2_S as a cardiac protectant is evaluated during the chemotherapy. Chegaev et al. found that doxorubicin has a more potent cytotoxicity on both doxorubicin-sensitive and doxorubicin-resistant osteosarcoma cells in the presence of H_2_S than that in the absence of H_2_S. The inhibitory effect of H_2_S on the P-glycoprotein in the doxorubicin-resistant osteosarcoma cells, resulting in the reduced efflux of doxorubicin and increased intracellular accumulation of DOXO, might be involved in the enhanced effectiveness of doxorubicin (Chegaev et al., 2016). Tesei and his colleague demonstrated that valproic acid induced an increase in cytotoxicity and mitochondria-dependent cell apoptosis but a decrease in invasive activity in cell lines of the non-small cell lung cancer (NSCLC) in the presence of H_2_S. Moreover, cisplatin caused an increased cytotoxicity on the NSCLC cells when it was administrated in the presence of an H_2_S-releasing valproate derivative ACS2. Also, they found that the cytotoxic effect of doxorubicin on the NSCLC cells was enhanced by the combined treatment with ACS2 (Tesei et al., 2012). The above mentioned studies suggested that co-treatment of H_2_S might enhance the anti-tumor of antineoplastic drugs, which further strengthened the clinical prospect of H_2_S as a cardiac protectant during the chemotherapy of tumor (Table 2).

**Table 2:** Original researches reports which stressed the effectiveness of anticancer drugs in the presence of H_2_S.

**References**	**Effectiveness of anticancer drugs**
Chegaev et al., 2016	Doxorubicin has a more potent cytotoxicity on both doxorubicin-sensitive osteosarcoma cells U-2OS and doxorubicin-resistant osteosarcoma cells U-2OS/DX30, U-2OS/DX100 and U-2OS/DX580 in the presence of H_2_S than that in the absence of H_2_S. The inhibitory effect of H_2_S on the P-glycoprotein in the doxorubicin-resistant osteosarcoma cells, resulting in the reduced efflux of doxorubicin and increased intracellular accumulation of DOXO, might be involved in the enhanced effectiveness of doxorubicin.
Tesei et al., 2012	1. Valproic acid induced an increase in cytotoxicity and mitochondria-dependent cell apoptosis but a decrease in invasive activity in cell lines of the non-small cell lung cancer (NSCLC) in the presence of H_2_S. 2. Cisplatin caused an increased cytotoxicity on the NSCLC cells when it was administrated in the presence of an H_2_S-releasing valproate derivative ACS2. 3. The cytotoxic effect of doxorubicin on the NSCLC cells was enhanced by the combined treatment with ACS2.

## Conclusion

H_2_S can play antioxidant, antiapoptotic and anti-inflammatory roles against chemotherapy-induced cardiotoxicity. H_2_S donors or synthetic releasing compounds have been verified to have the therapeutic potential for heart disorders. Particularly, the development of H_2_S-releasing DOXOs may become a new milestone for the treatment of cardiotoxicity among long-term cancer survivors.

## Author contributions

SD: wrote the manuscript; YH and HJ: revised the manuscript; TW: designed and supervised the writing of the paper; All authors approved the final version of the manuscript.

### Conflict of interest statement

The authors declare that the research was conducted in the absence of any commercial or financial relationships that could be construed as a potential conflict of interest.
